# Off-label use of an iliac branch device and a reversed iliac limb for a patient with a unilateral common iliac artery aneurysm and a narrow distal aorta: A case report

**DOI:** 10.1097/MD.0000000000032640

**Published:** 2023-01-13

**Authors:** Deokbi Hwang, Woo-Sung Yun, Hyung-Kee Kim, Seung Huh

**Affiliations:** a Division of Vascular and Endovascular Surgery, Department of Surgery, Kyungpook National University Hospital, Kyungpook National University School of Medicine, Daegu, South Korea.

**Keywords:** aneurysm, aortoiliac vessels, branched-stent-graft, common iliac artery, off-label use

## Abstract

Current bifurcated aortic endografts are unsuitable for patients with a narrow distal aorta except AFX2, which is unavailable in South Korea. An iliac branch device (IBD) was introduced to exclude iliac aneurysms while preserving the pelvic circulation. With advancements in endovascular techniques, various attempts for outside instructions for use have been reported to be practicable in certain patients.

**Patient concerns:**

A 58-year-old man was referred to our emergency room with an incidentally found left common iliac artery aneurysm (CIAA) in a general checkup.

**Diagnoses:**

Computed tomography angiogram showed a narrow distal aorta that tapered from 20 mm just below the renal artery to 13 mm at aortic bifurcation and a left isolated CIAA with a maximal diameter of 40 mm and 70 mm in length.

**Interventions:**

After left hypogastric artery embolization, the Cook IBD was placed at the aortic bifurcation, and the Bard Covera Plus stent-graft was deployed from the IBD cuff to the left external iliac artery. Then, a reversed Medtronic Endurant iliac limb was implanted into the infrarenal aorta down to the proximal IBD.

**Outcomes:**

The stent grafts were patent without endoleak at the 6-month follow-up.

**Lessons:**

In selected patients with an isolated CIAA with a narrow distal aorta, IBD can be used as a main body at the aortic bifurcation for successful aneurysm exclusion. However, considering the application of outside instructions for use, special attention and careful planning must be taken before the procedure.

## 1. Introduction

Despite inadequate anatomy for the manufacturer’s instructions for use (IFU), endovascular aneurysmal repair (EVAR) tends to be considered first for patients with increased surgical risk. Contrary to the traditional findings that emphasize the observance of IFU, it has been reported that IFU adherence no longer significantly affects EVAR outcomes.^[1,2]^ Accumulated experience and persistent attempts to overcome complex anatomy have achieved technical developments, including iliac branch devices (IBDs). Since its approval in 2006, IBD has widely been used for iliac aneurysm exclusion while maintaining pelvic circulation. The use of IBD within IFU showed excellent midterm outcomes in both bilateral and unilateral use.^[3]^ However, examples of outside IFU use have increasingly been reported in complex situations.^[4,5]^ We report a case of an isolated unilateral common iliac artery aneurysm (CIAA) and a tapered narrow aorta treated with concomitant off-label use of an IBD and a reversed iliac limb. We acquired informed consent from the patient and approval from our institutional review board.

## 2. Case report

**Figure 1.**
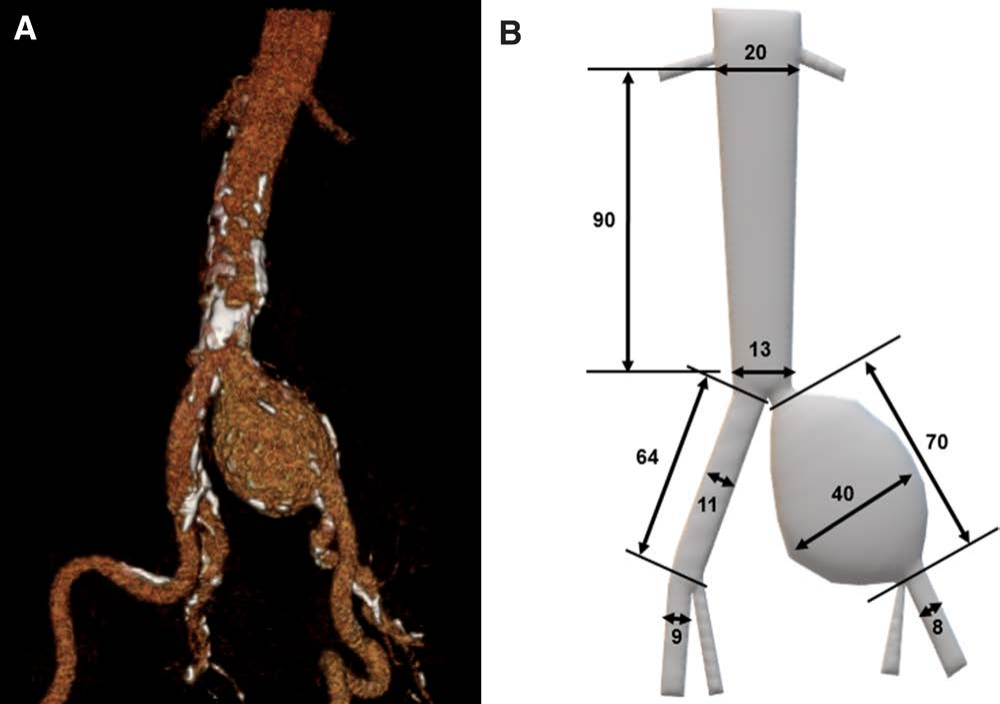
(A) Preoperative computed tomography angiography image with 3-dimensional reconstruction that shows a left common iliac artery aneurysm (LAO 35, CAU 15) and (B) a drawing using Paint 3D application in Accessories with detailed numerical values (unit, mm).

A 58-year-old man was referred to our emergency room with an incidentally found left CIAA in a general checkup. He had an approximately 10 cm-sized right reducible inguinal mass that had occurred 2 years prior to referral, and the patient was an hepatitis B virus carrier. On physical examination, his ankle and femoral artery pulses were palpable, and there was no abdominal tenderness. Except for a slightly elevated total cholesterol level (210 mg/dL), there were no laboratory abnormalities. Computed tomography angiogram showed a tapered and narrow distal aorta (20 mm just below the renal artery and 13 mm at aortic bifurcation) and a left isolated CIAA, which started just distal to the aortic bifurcation and showed a maximal diameter of 40 mm and 70 mm in length. It did not involve the internal iliac artery (IIA) (Fig. 1). Both IIAs were patent, but the ostium of the left IIA was narrow (4 mm).

**Figure 2.**
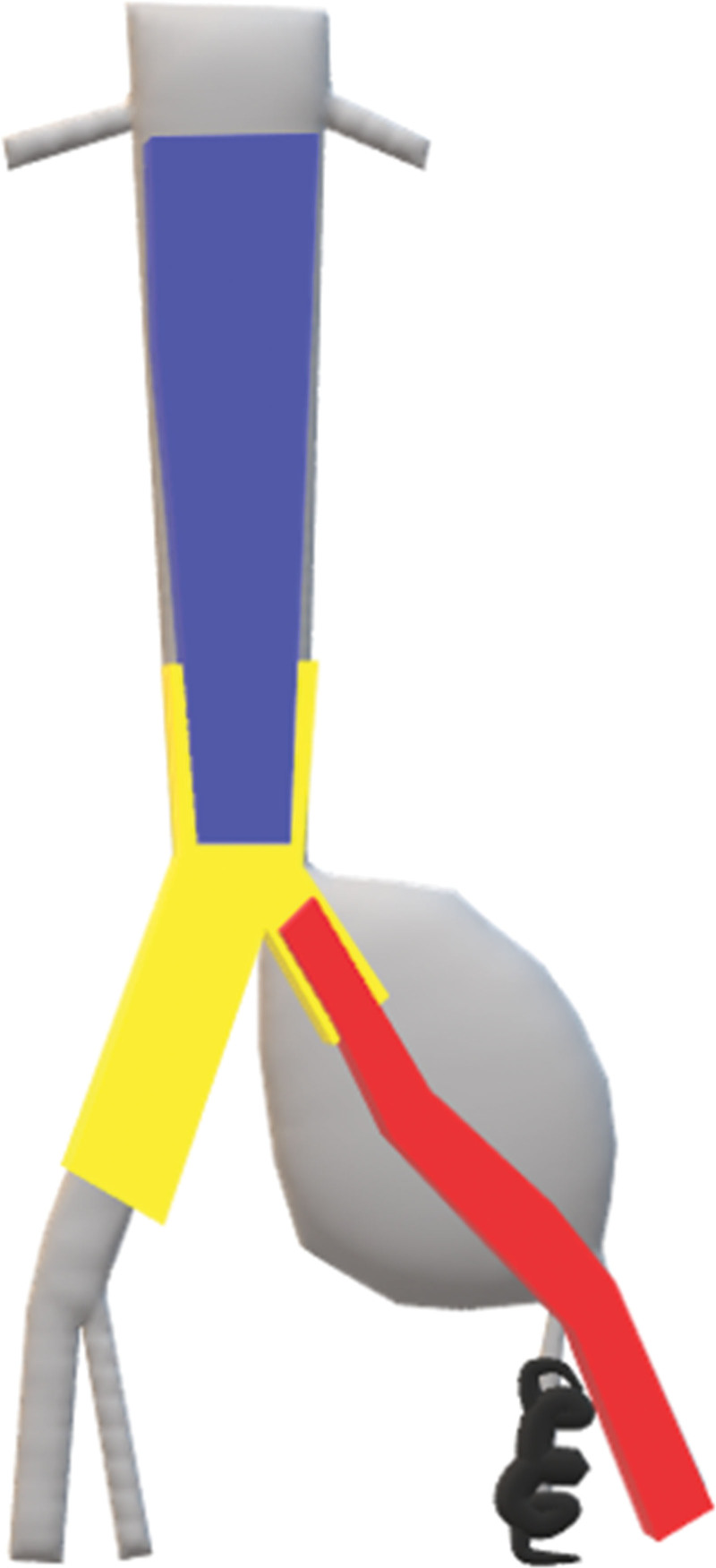
Preoperative planning with the equipment from 3 different manufacturers expected to be used. It was drawn in Paint 3D application (yellow, Cook IBD; blue, reversed Endurant iliac limb; red, Covera Plus stent-graft; black, Concerto microcoils). “Print color requested”.

**Figure 3.**
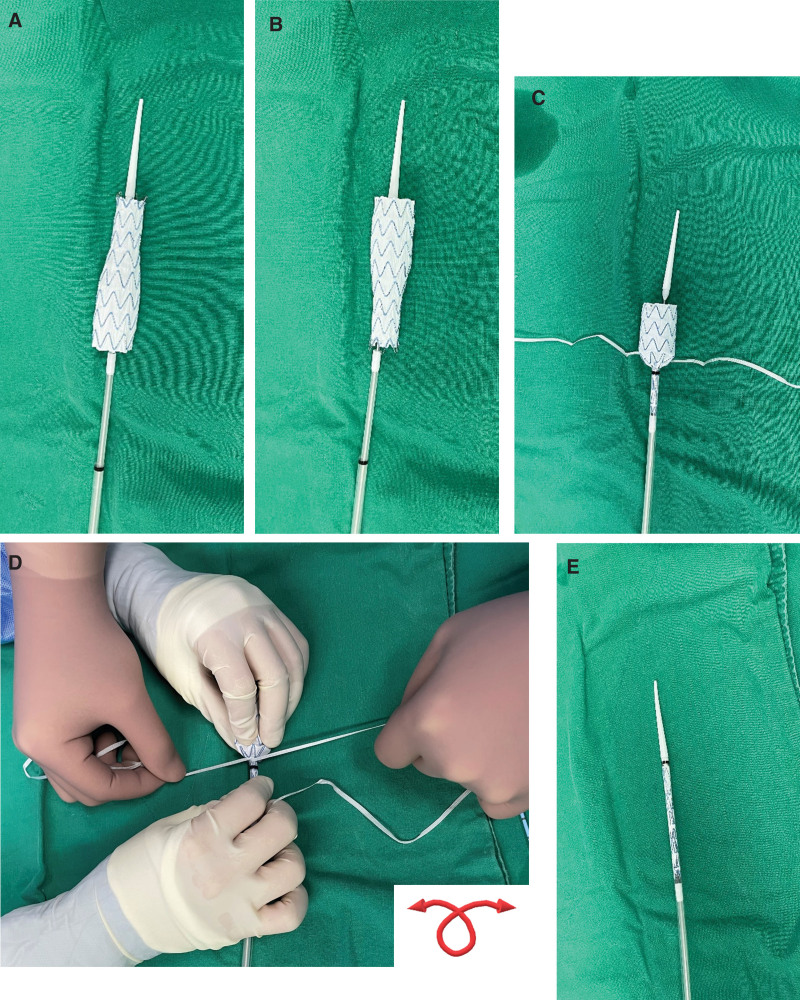
Benchwork steps of the preparation for proximal aortic extension with an Endurant iliac limb in an upside-down configuration. (A) Deploying a stent graft on a separate table aseptically. (B) Relocating the deployed stent graft in the opposite direction so that the released stent tapers downward with a proximal end of 24 mm and a distal end of 16 mm in diameter. (C) After half-mounted in the reverse direction. (D) Inserting the reversed endograft into the initially peeled sheath with the aid of nylon tape. One person collects the struts as much as possible by wrapping them with nylon tape, while the other holds both ends of the gathered struts with both hands and puts them one by one into the sheath so that the struts would not get caught in the tip. On the bottom right, the schematic drawing shows how the nylon tape is arranged. (E) After reloading a reversed limb in a 16 Fr delivery system.

Because AFX2 (Endologix Inc., CA) is unavailable in our country, there is no bifurcated mainbody suitable for a narrow distal aorta. Therefore, we decided to deploy an IBD at the aortic bifurcation. Figure 2 demonstrates the preoperative planning. As a proximal extension endograft to prevent type Ia endoleak, a 16 × 24 × 82 mm iliac limb stent-graft (Endurant IIs; Medtronic Vascular, Santa Rosa, CA) was used in an upside-down configuration. It was prepared in advance through a benchwork of unsheathing, direction change, and resheathing (Fig. 3).

**Figure 4.**
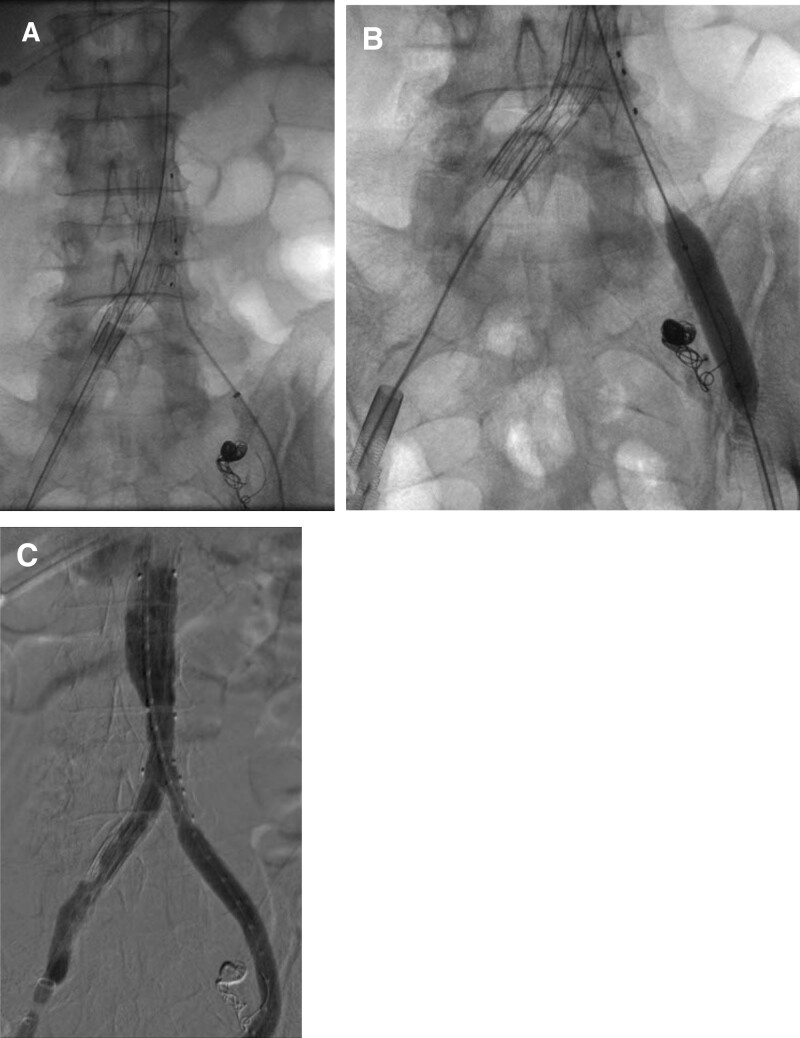
Endovascular procedure. (A) The advancement of the Cook iliac branch device (IBD) along the extrastiff wire from the right side and the placement of the side branch at approximately 10 mm above the aortic bifurcation. The left internal iliac artery (IIA) had just been embolized with microcoils. (B) The deployment of a 10 × 100 mm self-expanding covered stent from the left common femoral artery with an adequate overlapping zone, proximal to the IBD and distal to the external iliac artery, which was subsequently molded with a balloon catheter 10 mm in diameter. (C) After the proximal aortic extension to IBD with a reversed iliac limb, completion angiography shows a contrast filling defect in the right common iliac artery (partial) and the IIA (complete), suggesting thrombosis but no type I or III endoleak.

The endovascular procedure was as follows (Fig. 4). Access was acquired on both common femoral arteries (CFAs). First, ProGlide percutaneous closing devices (Abbott Vascular, Santa Clara, CA) were placed on 2 pieces on each side in the 10 and 2 o’clock directions. Eight and 10 Fr sheaths were inserted in the respective right and left sides. After embolization of the left IIA with 8 mm detachable microcoils (Concerto; Medtronic Inc., Minneapolis, MN) from the right CFA, a pigtail sizing catheter was inserted along the soft hydrophilic 0.035” guidewire (Aquatrack; Cordis Corp., FL) from the left side, and a 4 Fr Glide catheter (Terumo Corp., Tokyo, Japan) was inserted from the right side. After changing to an extrastiff wire (Lunderquist; Cook Medical, Bloomington, IN), a Zenith Bifurcated Iliac Side® (12-45-41) IBD (Cook Medical, Bloomington, IN) was advanced from the right side, and its side branch was placed approximately 10 mm above the aortic bifurcation. Following selection of the IBD orifice from the left CFA, a 10 × 100 mm self-expanding covered stent (Covera Plus; Bard Peripheral Vascular Inc., Murray Hill, NJ) was deployed, obtaining a respective 20 mm overlapping zone proximally with IBD and distally with an intact external iliac artery (EIA). Then, proximal extension to IBD was performed with a prepared reversed iliac limb. Following balloon molding, completion angiography showed no type I or III endoleak but did show a contrast filling defect in the right CIA and IIA, suggesting thrombosis. We decided to wait and watch while prescribing the patient anticoagulation therapy. After completing the initially placed ProGlide knots, the patient subsequently underwent an open repair of the right inguinal hernia.

**Figure 5.**
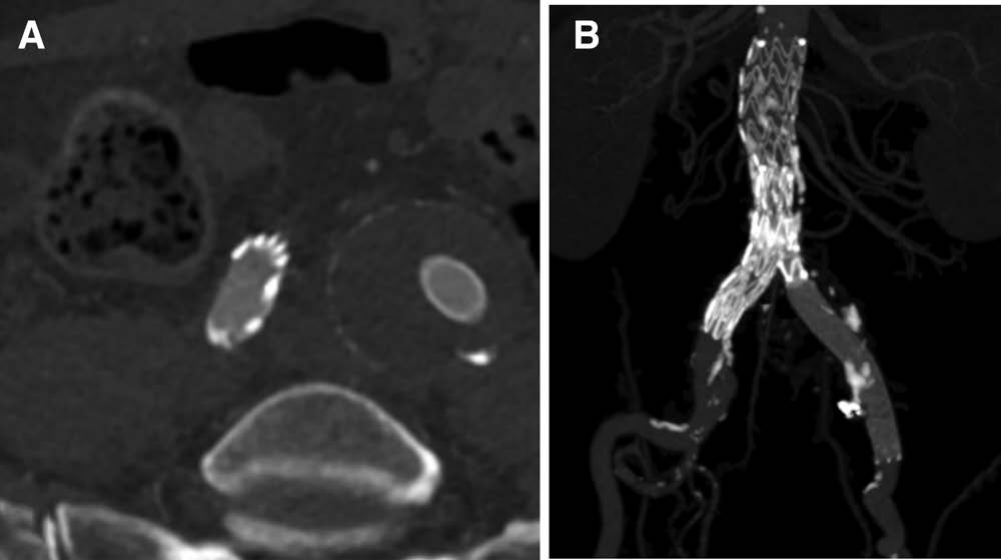
Follow-up computed tomography image after a 1-month anticoagulation with warfarin (target INR 2–2.5) shows (A) complete resolution of the thrombosis in the right common iliac artery and (B) no evidence of endoleak on maximum intensity projection with 3-dimensional reconstruction.

On postoperative day 7, follow-up computed tomography angiogram showed an obvious partial thrombus on the right CIA; however, the patient’s ankle pulse was strongly palpable. After 1 month of anticoagulation with warfarin (target INR 2–2.5), the thrombus was completely resolved, and there was still no endoleak (Fig. 5). Thereafter, the patient was switched from warfarin to aspirin. At 6 months after the operation, ultrasound showed no abnormal findings, and he was doing well with gradual improvement of buttock claudication that must have been induced by IIA embolization.

## 3. Discussion

We depicted the successful treatment of an isolated CIAA accompanied by a tapered distal aorta with concomitant off-label use of Zenith Bifurcated Iliac Side IBD and Endurant IIs iliac limb. This case demonstrates the feasibility of IBD use at the aortic bifurcation as an alternative to standard EVAR in certain anatomies. The Cook device was the only available IBD in South Korea. Although the manufacturer’s IFUs recommend proximal extension in the infrarenal aorta, it has been reported that isolated CIAAs can be treated effectively and safely with IBD alone.^[6]^ These examples applied IBDs at the iliac bifurcation while keeping the IFU. However, in this case, the proximal CIA was too short to be safely sealed with IBD alone at the iliac bifurcation.

A strict protocol of IBD states an aneurysmal change in a distal IIA as an absolute contraindication and heavy iliac tortuosity and wide angle as relative contraindications.^[7]^ After replacing IIA and CIA with CIA-EIA and distal aorta, respectively, the applicability of IBD at aortic bifurcation was confirmed without violating the above conditions.

Although the standard IFU of Cook IBD recommends the use of balloon-expandable stent-graft (BESG) as a bridging stent in an up-and-over manner, in this case, 1 long self-expanding stent-graft (SESG) was retrogradely introduced on the ipsilateral side of the aneurysm and could be correctly placed. We also decided to use 1 long SESG instead of many pieces of BESG to reduce the possibility of endoleak. Lima et al reported similar outcomes of iliac aneurysm repair between SESG and BESG with respect to patency, buttock claudication, and reintervention rates. However, possibly due to the more frequent use of BESGs outside the IFU than SESGs, BESGs showed a higher percentage of distal instability and required more stents than SESGs.^[8]^

Various methods were considered preoperatively. As an endovascular option, conventional EVAR is difficult because of potential limb occlusion by limb competition within the narrow distal aorta. Additionally, kissing stent grafts within aortic tube grafts were excluded because of possible gutter leakage as well as limb competition. As a hybrid option, aorto-uni-iliac stent-graft and subsequent femorofemoral bypass were excluded considering a relatively young age and extra-anatomical configuration. Additionally, EIA to IIA bypass could be followed by standard EVAR. However, we performed an endovascular option because of the patient’s strong willingness to avoid laparotomy. Meanwhile, IBD deployment was expected to be difficult because of insufficient space in the distal aorta and contralateral CIA. Therefore, we chose the shortest length of IBD.

There have been some reports on the off-label use of IBD at the aortic bifurcation in patients with a CIAA with a kinked aortic graft or with a narrow distal aorta.^[9–11]^ However, regarding off-label use, successful cases seem to be preferentially reported compared with failed cases. Although it is still too early to conclude that IBD is safe and efficacious in most cases outside the IFU, versatile applications can be attempted under long-term surveillance.

In addition to IBD, the off-label use of flared endografts has often been reported in patients with a tapered aorta or iliac artery.^[12,13]^ According to the IFU of Cook IBD, a bridging stent-graft with a distal diameter of 16 mm is required between the proximal aortic graft and IBD. Considering 15 to 20% oversizing, the aortic graft with a proximal diameter of 23 to 24 mm is adequate because the aortic diameter is 20 mm just below the renal artery. An aorto-uni-iliac stent-graft with a proximal diameter of 25 mm and a minimum length of 102 mm was excluded since it could cover the IBD cuff opening in the infrarenal aorta with a length of 90 mm. Hence, a reversed Endurant iliac limb of 82 mm in length was selected because it could provide an overlapping zone of 30 to 40 mm while preserving the IBD patency.

Intraoperatively, there were some unmentioned procedural mistakes. We were under a delusion about the opening site of the distal tip of the side branch. Numerous attempts to pass a wire using various catheters through the wrong site of stent-graft for quite some time seemed to have resulted in thrombosis as well as additional operation hours. Fortunately, we realized the mistake and were able to proceed with the operation as planned, albeit belatedly.

## 4. Conclusion

In conclusion, in selected patients with an isolated CIAA with a narrow distal aorta, IBD can be used as a main body at the aortic bifurcation for successful aneurysm exclusion. However, considering the application of outside IFU, special attention and careful planning must be taken before the procedure.

## Author contributions

**Conceptualization:** Deokbi Hwang, Woo-Sung Yun.

**Data curation:** Deokbi Hwang, Hyung-Kee Kim, Seung Huh.

**Formal analysis:** Deokbi Hwang.

**Investigation:** Deokbi Hwang, Woo-Sung Yun.

**Methodology:** Woo-Sung Yun, Hyung-Kee Kim, Seung Huh.

**Supervision:** Woo-Sung Yun, Hyung-Kee Kim, Seung Huh.

**Validation:** Hyung-Kee Kim, Seung Huh.

**Visualization:** Deokbi Hwang.

**Writing – original draft:** Deokbi Hwang.

**Writing – review & editing:** Woo-Sung Yun.
